# Isolation and characterization of a main porin from the outer membrane of *Salinibacter ruber*

**DOI:** 10.1007/s10863-022-09950-7

**Published:** 2022-10-13

**Authors:** Domenica Farci, Emma Cocco, Marta Tanas, Joanna Kirkpatrick, Andrea Maxia, Elena Tamburini, Wolfgang P. Schröder, Dario Piano

**Affiliations:** 1 Department of Chemistry, Umeå University, Linnaeus väg 6, 90736 Umeå, Sweden; 2 Laboratory of Plant Physiology and Photobiology, Department of Life and Environmental Sciences, Università degli Studi di Cagliari, V.le S. Ignazio da Laconi 13, 09123 Cagliari, Italy; 3 The Francis Crick Institute, 1 Midland Road, NW1 1AT London, UK; 4 Laboratory of Economic and Pharmaceutical Botany, Department of Life and Environmental Sciences, Università degli Studi di Cagliari, V.le S. Ignazio da Laconi 13, 09123 Cagliari, Italy; 5 Department of Biomedical Sciences, Università degli Studi di Cagliari, Cittadella Universitaria sp. 8, 09042 Monserrato, CA Italy

**Keywords:** Cryo-EM, Carotenoids, Halophile bacteria, Outer membrane, Outer membrane proteins, Cell envelope, Mass spectrometry, *Salinibacter ruber*

## Abstract

*Salinibacter ruber* is an extremophilic bacterium able to grow in high-salts environments, such as saltern crystallizer ponds. This halophilic bacterium is red-pigmented due to the production of several carotenoids and their derivatives. Two of these pigment molecules, salinixanthin and retinal, are reported to be essential cofactors of the xanthorhodopsin, a light-driven proton pump unique to this bacterium. Here, we isolate and characterize an outer membrane porin-like protein that retains salinixanthin. The characterization by mass spectrometry identified an unknown protein whose structure, predicted by AlphaFold, consists of a 8 strands beta-barrel transmembrane organization typical of porins. The protein is found to be part of a functional network clearly involved in the outer membrane trafficking. Cryo-EM micrographs showed the shape and dimensions of a particle comparable with the ones of the predicted structure. Functional implications, with respect to the high representativity of this protein in the outer membrane fraction, are discussed considering its possible role in primary functions such as the nutrients uptake and the homeostatic balance. Finally, also a possible involvement in balancing the charge perturbation associated with the xanthorhodopsin and ATP synthase activities is considered.

**Supplementary Information:**

The online version contains supplementary material available at 10.1007/s10863-022-09950-7.

## Introduction

*Salinibacter ruber* is a halophilic bacterium (Antón et al. 2002; Oren 2013) that is well known for its characteristic ability to produce ATP through a light-driven proton gradient across the membrane (Balashov and Lanyi 2007). The mechanism by which the ATP synthesis takes place is mediated by retinal as for all type-1 rhodopsins (Kanehara et al. 2017). The bacteriorhodopsin type-1 is very peculiar in several aspects, in particular it represents a rare case where a light-harvesting antenna is coupled with the bacteriorhodopsin’s retinal and binds salinixanthin, a carotenoid capable of light harvesting and excitonic energy transfer to the retinal (Balashov et al. 2005, 2006; Boichenko et al. 2006; Imasheva et al. 2006). Thus, in this bacteriorhodopsin, called xanthorhodopsin (XR), the salinixanthin complements in quality and quantity the spectral properties of the retinal, resulting in an increased quantum yield and efficiency of this light-harvesting/proton-pumping machine (Boichenko et al. 2006; Balashov et al. 2008). Due its crucial importance, the XR complex is present in high amounts in the cell envelope of *S. ruber* and provides a specific and characteristic function to it.

In bacteria, the cell envelope is also characterized by a specific set of porins that regulates the cell trafficking with the environment and actively contributes to maintain and sustain the cell homeostasis (Vergalli et al. 2020; Farci et al. 2022). It is reasonable to expect the presence of a similar set-up existing also in *S. ruber*. Halobacteria are characterized by a complex inward/outward trafficking of ions, thus they need to cope with potential conditions of compromised homeostasis that might arise. This is particularly true for *S. ruber*, in which there is also the need of a complementary system aimed at balancing the perturbation associated with the XR activity. Here, we have isolated and characterized a main protein of the *S. ruber* cell envelope, describing its properties by different means. When characterized by Mass Spectrometry (MS), this ~ 20 kDa protein was identified as an unknown protein carrying an Outer Membrane Protein (OMP) beta-barrel domain. The protein appears to be pigmented due to the non-covalent retention of the carotenoid salinixanthin and its structure is predicted by Artificial Intelligence (AI) to consist of a 8 strands beta-barrel transmembrane organization typical of porins. Consistently, cryo-EM micrographs showed significant elements of coincidence in shape and dimensions with the ones predicted by AI. The protein is found to be part of a functional network clearly involved in the trafficking of nutrients and in the homeostatic balance. Similarly to XR, the significant presence of this protein in the cell envelope and the retention of salinixanthin might suggest a functional relationship between this OMP and the XR system in coincidence with its involvement in primary functions such as nutrients uptake and homeostatic balance. Considering the sieve function played by the large variety of porins in OMP systems, results are discussed in terms of an outer membrane’s functionalization able to deal with the peculiar autotrophy and the extreme environment into this bacterium have to sustain the nutrients uptake.

## Materials and methods

### Cell culturing

*Salinibacter ruber* strain M31 (DSM 13,855) was purchased from DSMZ (German Resource Center for Biological Material, Braunschweig, Germany). The strain was grown at 37 °C in a Modified Growth Medium (MGM; 5% Peptone, 1% Yeast extract) with 23% total salt (3.1 M NaCl, 170 mM MgCl_2_ ⋅ 6H_2_O; 38.5 mM MgSO_4_ ⋅ 7H_2_O; 67 mM KCl; 2.9 mM NaHCO_3_; 0.62 mM NaBr) (Smith 2009). Bacterial cells were harvested in the late exponential growth phase (OD_600_ = 1.25) by centrifugation at 5000 × *g* for 10 min at 4 °C and resuspended in 50 mM Sodium phosphate buffer pH 7.8.

### Cell envelopes isolation

Cell envelopes were purified at 4 °C accordingly to Farci et al. (2014). The resuspended pellet was treated with 100 U DNase I (DNase I recombinant, RNase-free Roche) and disrupted using a French Pressure Cell, subsequently, the unlysed cells were removed by low-speed centrifugation for two times at 5000 × *g* for 10 min at 4 °C. The final supernatant was centrifuged again at 48,000 × *g* for 10 min at 4 °C and the red pellet, consisting of cell envelope fragments, was resuspended in 10 mL of Sodium phosphate buffer (50 mM Sodium phosphate pH 7.8). The main protein component was isolated as reported in Farci et al. (2014) (patent WO2017125884 (A1) – 2017-07-27) with minor modifications. Briefly, to remove surface polysaccharides, the membrane suspension was incubated under agitation (800 rpm) with 100 µg/mL lysozyme for 8 h at 25 °C. This treatment led to the selective release in solution of a main protein component. The sample was subsequently centrifuged (48,000 × *g* for 10 min at 4 °C) to clarify the released protein from the residual suspension.

Differently from previous reports (Farci et al. 2014) (patent WO2017125884 (A1) – 2017-07-27), the second disruption step by French Pressure Cell was not required due to the absence in *S. ruber* of an S-layer, which confers great robustness to the cell envelope (Farci et al. 2014, 2021).

### Protein purification

The obtained supernatant was concentrated using a Vivaspin 20 ultrafiltration membrane with a 100-kDa cutoff (GE Healthcare) to a volume of ~ 150 µL. After concentration, a final volume of 100 µL was loaded on a Size Exclusion Chromatography (SEC) column (Superose 6 10/300GL, GE Healthcare) previously equilibrated in 50 mM Sodium phosphate buffer pH 7.8 at flow rate of 0.5 mL/min. The main peak was pooled, and the molecular weight was estimated using a molecular marker (Gel Filtration Standard, Biorad). For the experiments with detergent, immediately after lysozyme treatment, the supernatant was subject to a mild solubilization using 0.05% (w/v - final concentration) of n-Dodecyl-β-Maltoside (β-DDM) for 5 min at 4 °C. After solubilization, the sample was centrifuged for 5 min at 10,000 x g. The SEC experiments for these samples were performed using 50 mM Sodium phosphate buffer pH 7.8 added with 0.05% of β-DDM. In these studies, all chromatography columns were subjected to the ReGenFix procedure (https://www.regenfix.eu/) for regeneration and calibration prior use (Farci et al. 2021).

### Absorption spectroscopy

The absorption spectroscopy measurements were performed on a Pharmacia Biotech Ultrospec 4000 spectrophotometer at 4 °C in the range of 200–800 nm with an optical path length of 1 cm (quartz cell, Hellma Analytics). Spectra were recorded at a protein concentration of 0.05 mg ml^− 1^.

### Polyacrylamide gel electrophoresis (PAGE)

Sodium Dodecyl Sulphate-Polyacrylamide Gel Electrophoresis (SDS-PAGE) with a 10% (w/v) separating and a 4% (w/v) stacking gel was used (Farci et al. 2014). Both samples, cell envelope patches and the main SEC peak, were denatured with Rotiload (Roth), boiled for 5 min and centrifuged for 5 min before loading. After the electrophoretic separation, the gels were stained with Coomassie Brilliant Blue G-250.

### Mass spectrometry, structural prediction, and bioinformatic analysis

The gel bands were excised from SDS-PAGEs and digested with trypsin prior to further processing (Farci et al. 2014). Data analysis was performed with MaxQuant software (version 1.0.13.13), and the obtained files were used with Andromeda (Cox et al. 2011) against a species-specific database. The structural prediction for the main protein component (Uniprot entry Q2S6E7) was done by using the AI prediction method of AlphaFold (https://github.com/deepmind/alphafold) (Jumper et al. 2021). The signal peptide prediction was calculated using TISIGNER (https://tisigner.com). The interactions and related network for the SRU_0081 (Q2S6E7) were analyzed using STRING (https://string-db.org) with a minimum required interaction score of 0.4 and a maximum number of 5 interactions for the first shell excluding the second shell. Finally, for each entry identified by MS, the function was assigned using the InterPro EMBL-EBI Server (http://www.ebi.ac.uk/interpro/) (Jones et al. 2014; Mitchell et al. 2015), and the subcellular localization was assessed by using the CELLO v.2.5 software (http://cello.life.nctu.edu.tw/) (Yu et al. 2006).

### Cryo-electron microscopy

Quantifoil R2/2 holey carbon grids were glow-discharged prior use. Both samples, the isolated cell envelopes and the isolated protein, were blotted and vitrified using a Vitrobot plunge-freezing machine (Mark IV, ThermoFisher) at room temperature (blot force 0, blotting time 3 s, 100% humidity), and placed in autogrid (FEI, Eindhoven, Netherlands) prior image acquisition.

Micrographs were acquired with a Titan-Krios TEM (ThermoFisher) at an operating voltage of 300 kV and equipped with a Cs-corrector (cs 2.7 mm), a Quantum GIF energy filter (slit width set to 20 eV), and a post-GIF K2 camera (Gatan) in counting mode. Images were manually recorded by the EPU software at a nominal magnification of 33.000×, yielding a final image pixel size of 4.37 Å. Image defocus was set at − 3.0 μm, the total electron dose used to acquire a single image was ~ 40 electrons/Å^2^.

## Results

### The red-pigmented cell envelope of *S. ruber* consists of a characteristic pattern of proteins

**Fig. 1:**
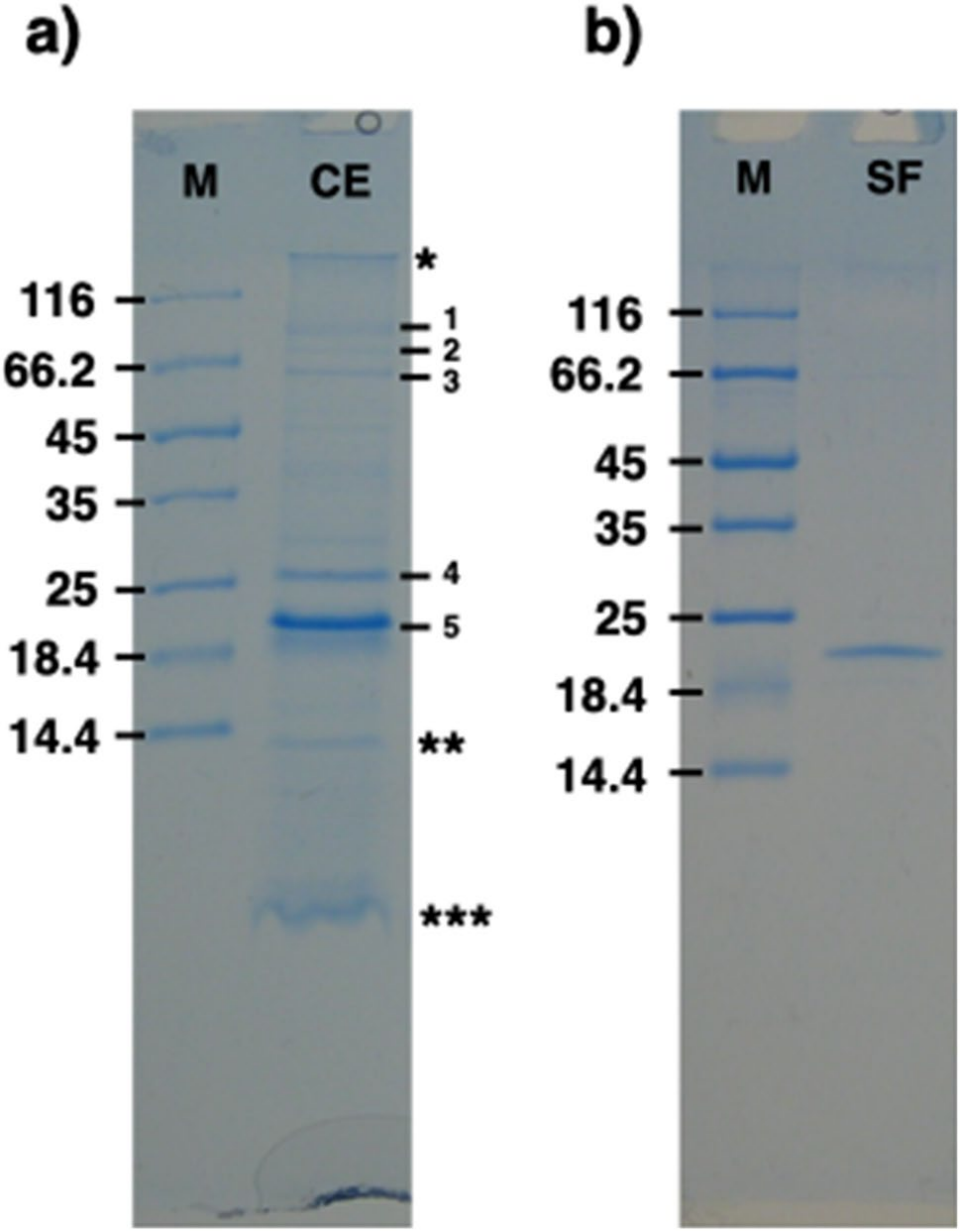
**a **SDS-PAGE of the isolated cell envelope fraction. When characterized by SDS-PAGE, the cell envelope samples resolved in a typical pattern of five bands. The lane labelled with CE indicates the cell envelope samples, and the main bands are numbered from 1 to 5, with the bands 4 and 5 being the most represented; the asterisks indicate the running/stacking gel interface (*), the lysozyme band used for the peeling procedure (**), and the separation front with pigments (***), respectively. The lane labelled with M indicates the molecular marker. The sample shown here is the total cell envelope pool immediately after lysozyme digestion and before any further step; **b **SDS-PAGE of the selectively released fraction. The selectively released fraction (SF) of the cell envelope pool was collected by centrifugation after lysozyme digestion and washed/concentrated with a 100 kDa cutoff concentrator. This procedure led to a complete removal of lysozyme. The selectively released fraction resolved in a single band (lane SF). The lane labelled with M indicates the molecular marker. The gels are cropped for clarity purpose

After isolation, the cell envelope’s homogeneity was assessed by electron microscopy (Sup. Fig. 1) and its protein composition was analysed by denaturing gel electrophoresis (SDS-PAGE). The samples resolved into a typical pattern of five protein bands, of which two were dominant components with an apparent mass of ~ 20 and ~ 26 kDa (Fig. 1a). Furthermore, as common for this type of samples, the cell envelopes retained a typical violet-pink colour indicating the presence of a pigmented cofactor.

### A violet/pink-pigmented protein is a dominant component in the cell envelope of *S. ruber*

**Fig. 2:**
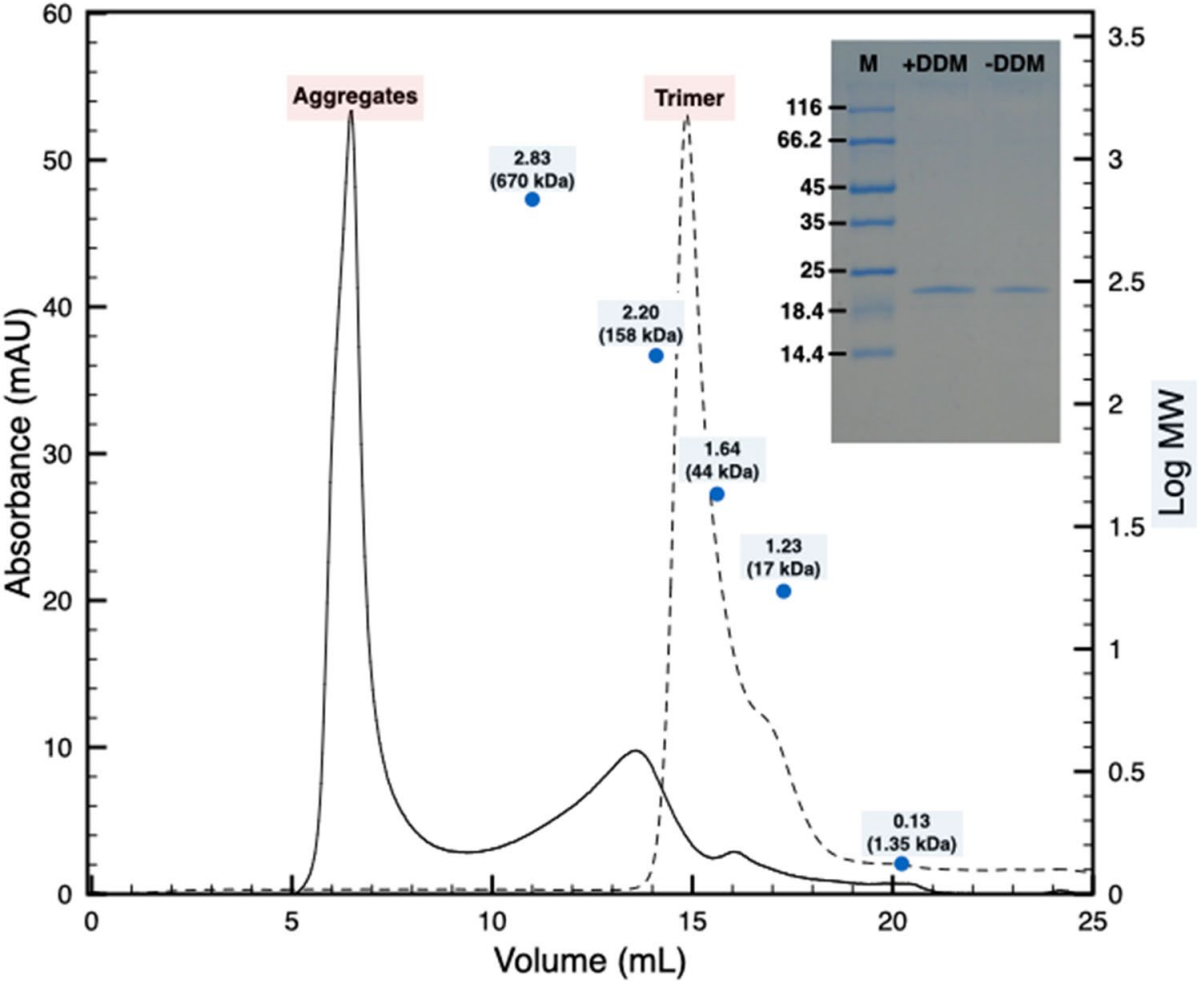
SEC profiles of the selectively released cell envelope fraction in absence and presence of DDM. In absence of DDM the protein samples appear characterized by two peaks a main one at high molecular weight (apparent mass > 800 kDa) and other secondary peaks at lower apparent molecular masses (continuous line). In presence of β-DDM the protein samples resolve into a single peak at an apparent mass of ~ 60 kDa followed by a shoulder (dashed line). The SDS-PAGE of the main peak for the SEC without DDM (-DDM) and with DDM (+ DDM) are also shown; the lane labelled with M indicates the molecular marker (Fig. 2, inset). The blue dots and the associated number represent the Log Molecular Weight (mass) of the molecular marker expressed in kDa. The gel is cropped for clarity purpose

A subsequent step of lysozyme digestion before solubilization unexpectedly led to the release in solution of the main protein component of ~ 20 kDa and a few faint secondary bands (Fig. 1b). Given the significant level of purity, we assessed the oligomeric state of the protein in solution by Size Exclusion Chromatography (SEC). The sample resolved into a reproducible profile with a dominant peak at an apparent mass higher than 800 kDa and showed the presence of secondary peaks at lower apparent molecular masses (Fig. 2). The SDS-PAGE characterization of the main SEC peak confirmed it as being composed of the dominant 20 kDa protein, and, because of its elution in the void range of the SEC, suggested it to be most likely an aggregate of the protein (Fig. 2, inset). To evaluate these hypotheses, considering the membrane-related origin of these samples, a step of mild solubilization using 0.05% β-DDM was introduced before the SEC step and, consistently, the SEC was performed in a buffer integrated with 0.05% β-DDM. This experiment led to a single peak at an apparent mass of ~ 60 kDa followed by a shoulder (Fig. 2) and the SDS-PAGE confirmed it to be composed only by the dominant 20 kDa protein (Fig. 2, inset). Similar results are also observed in non-concentrated samples resolved by SEC in absence of detergent (data not shown) and in diluted samples used for cryo-EM (see next paragraph). These results suggested a state of reversible aggregation for concentrated samples resolved by SEC in absence of detergent. The observation of a reversible protein aggregation, especially for short periods and before precipitation occurrence, is not unusual and has been also associated to regulation and homeostatic mechanisms (Saad et al. 2017). In the detergent treated samples, the identified apparent mass of ~ 60 kDa might speak for a trimeric complex, also typical for this type of proteins: however, due to the peculiar behavior of the protein, structural studies would be more appropriate to confirm this hypothesis. Finally, both isolated samples, without and with β-DDM, retained the typical violet-pink colour suggesting the specific retention of a cofactor, which was further investigated by absorption spectroscopy.

### Mass spectrometry analysis identifies the main OM constituent as an unknown outer membrane protein

Mass spectrometry analysis identifies the main OM constituent as an unknown outer membrane protein

Further analyses by MS were performed on the cell envelope samples and the isolated protein. The analysis was performed on the pattern of five main bands shown in Figs. 1a and 2, allowing the identification of the main protein components for each band and providing hints about their possible roles in the outer membrane and the cell envelope. In particular, this analysis identified the lighter and main ~ 20 kDa band (Fig. 1a, band 5), which is shared between the cell envelope samples and the isolated SEC sample (Fig. 2), as the uncharacterized protein with Uniprot entry Q2S6E7 (Table 1). This protein has an Outer Membrane Protein_β-barrel domain that allows to localize it in the outer membrane and identify it as a possible porin, as also suggested by the structural prediction (see next paragraph). The other four bands, which are exclusive of the cell envelope samples, are secondary components at about 25 kDa and above 66 kDa (Fig. 1a, bands from 1 to 4). These bands were identified as the Uniprot entries Q2S1A3 (Band 4), Q2S257 (Band 3), and Q2RZJ5 (Bands 2 and 1), which are an uncharacterized DUF5017 domain-containing protein, a Dihydrolipoyl dehydrogenase, and TonB-dependent receptor domain protein, respectively (Table 1). Further bioinformatic analyses allowed the possible localizations and functions of these entries, in particular, the presence of a TonB-dependent receptor (Q2RZJ5) provided further evidence for the OM origin and the porin-related network of the sample components (Table 1).

**Table 1:** Identification of the main cell envelope components by Mass Spectrometry

SDS-PAGE band	Uniprot identifier	Protein name	Mass (kDa)	Biological process	Localization prediction
band 1	Q2RZJ5	TonB-dependent receptor domain protein	91.413	Interaction with porins to regulate active transport	Outer Membrane
band 2	Q2RZJ5	TonB-dependent receptor domain protein	91.413	Interaction with porins to regulate active transport	Outer Membrane
band 3	Q2S257	Dihydrolipoyl dehydrogenase	52.507	Cell redox homeostasis	Cytoplasmic
band 4	Q2S1A3	DUF5017 domain-containing protein	29.177	Uncharacterised	Extracellular
band 5	Q2S6E7	OMP_b-brl domain-containing protein	22.196	Omp-like activity	Outer Membrane

The table shows the main components isolated from the cell envelope of *S. ruber*. Information about biological process and predicted localization for each protein are provided

### The isolated protein is part of an OMPs-network, is predicted to be a porin, and displays a typical shape

The isolated protein is part of an OMPs-network, is predicted to be a porin, and displays a typical shape

**Fig. 3:**
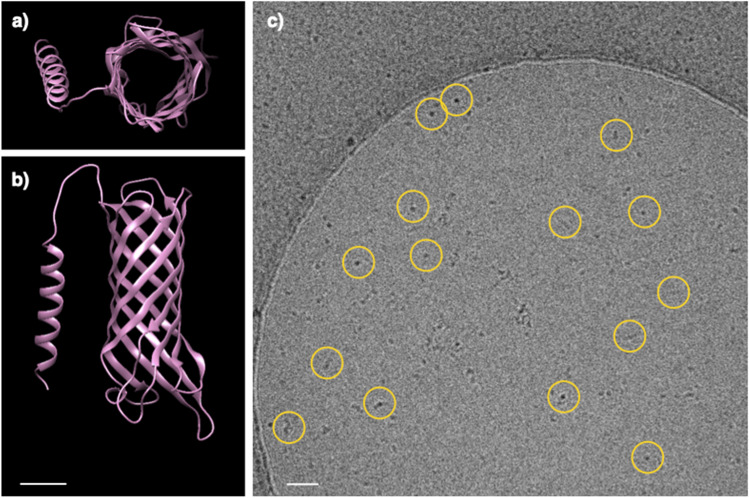
Structure prediction and cryo-EM micrograph of the OMPb-like protein. The structure of the OMPb-like protein as predicted by Alpha-Fold. In (**a**), a top view with a detail of the pore is shown; in (**b**), a side view displaying a typical porin-like structure with a 8 stranded β-barrel organization is shown. The α-helix represents the signal peptide region. A typical grid field of the isolated protein visualized by cryo-EM (**c**). Several orientations of the particle are shown (yellow rings) including evident top views (yellow rings with dark dots). The particle is typically lengthened with one dimension dominating on the other two. The scale bar in (**a**) and (**b**) indicates 10 Å, while the scale bar in (**c**) indicates 100 Å

The dominant protein Q2S6E7, hereinafter called the OMPblike protein because of its Outer Membrane Protein_β-barrel domain, was further characterized bioinformatically and by cryo-EM. The OMPb-like structure was predicted by AlphaFold (Jumper et al. 2021) as a typical porin-like structure with an 8 stranded β-barrel organization and a separated α-helix, the latter independently predicted by TISIGNER to be the signal peptide region from Met-1 and Ala-29 (Fig. 3a and b). Furthermore, the software STRING was used to confirm the functional context and the localization of the OMPb-like. This analysis showed how the main functional “neighboring” proteins of this OMPb-like are all OMPs, hence porins or transporters involved in the nutrient binding and uptake (Table 2, inset).

**Table 2.**
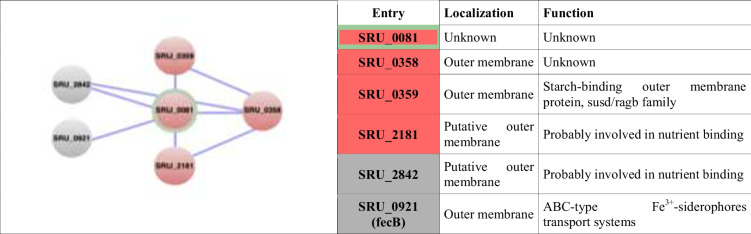
Identification of the OMPb-like protein’s functional neighbours and network

The table shows the five closest proteins in the OMPb-like protein’s network and the picture shows a scheme of the hierarchic connections. In red-green is shown the OMPb-like protein, the three proteins clustered in red are the closest, the two grey proteins are less connected

Finally, the isolated samples were visualized by cryo-EM. Micrographs showed a dominant particle with a typical elongated shape, thus having one dimension dominating on the other two (round-dot shape) (Fig. 3b). These experiments also showed a proper monodispersity and the absence of aggregates in the samples, both important indicators of its stability. Noteworthy, when measured, the lengths of the predicted structure and the one of the side views in the micrographs are comparable (~ 60 Å) and in the range of the typical outer membrane thickness.

### The isolated protein binds the carotenoid salinixanthin

**Fig. 4:**
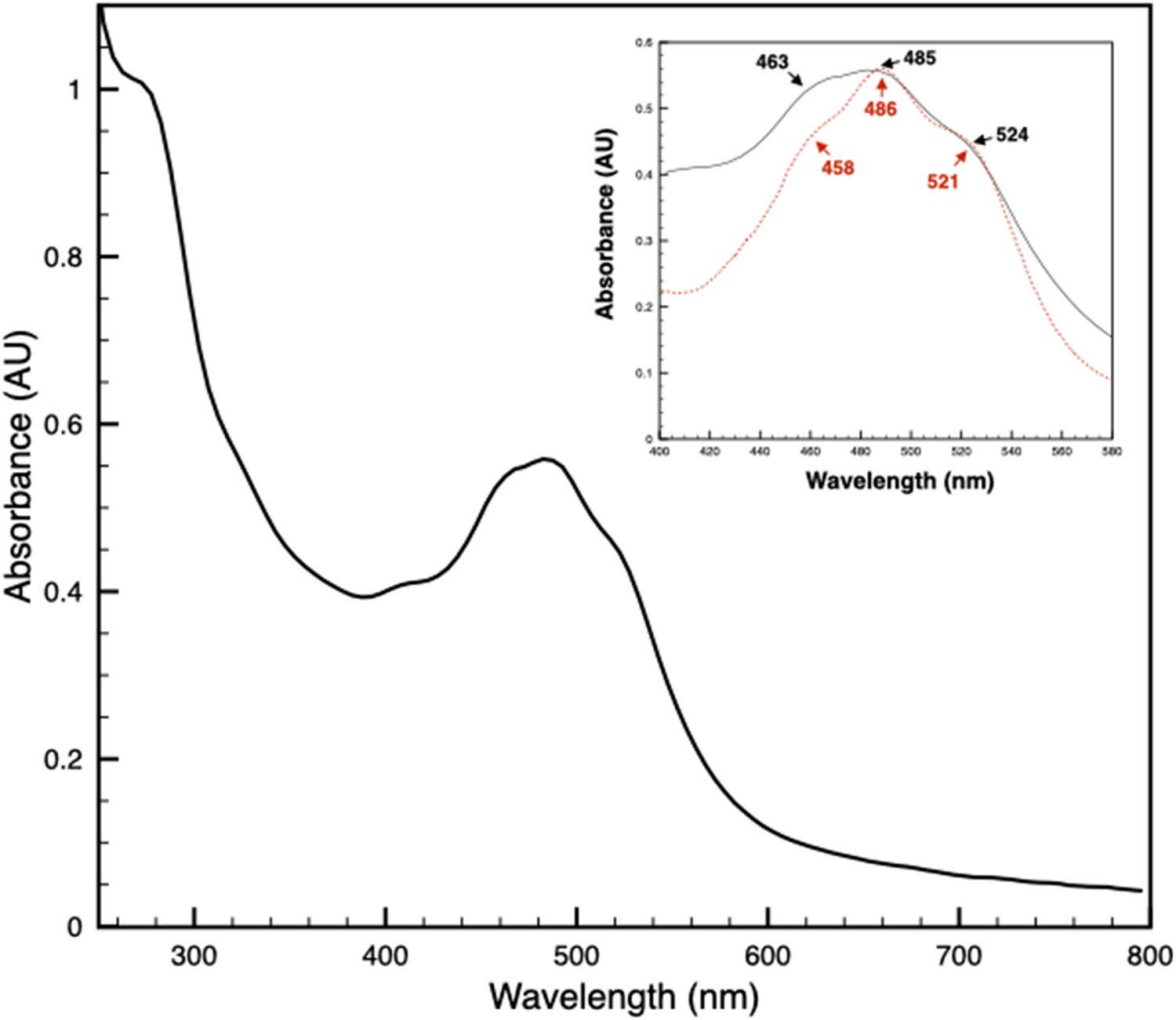
Characterization of the sample by UV-Vis absorption spectroscopy**. **OMPb-like samples showed a typical and intense absorption in the UV range related with the protein backbone. In the visible region, it is evident the “three-fingers” trace related to the polyene-absorbing region typical for carotenoids. The inset shows a detail of the visible region with three bands at 463, 485, and 524 nm (black dashed line) and its comparison with the salinixanthin profile shown by Lanyi and Balashov (2008) (red dotted line)

Finally, the typical violet-pink colour of the OMPblike samples suggested the retention of some cofactor that is most likely represented by one of the typical carotenoids of this bacterium. After isolation by SEC (without and with β-DDM), the selective retention of the carotenoid by the protein was assessed empirically during protein concentration by observing the absence of colour in the flow through during concentration steps. Furthermore, a direct extraction of the pigment from the protein was achieved by treating the sample with 80% acetone, indicating the carotenoid to be bound by non-covalent interactions (data not shown). Absorption spectroscopy in the UV-Vis range at room temperature of the samples isolated in absence of detergent showed a “three-fingers” trace related to the polyene-absorbing region, which is typical for carotenoids (Fig. 4). The two bands at 463 and 485 nm and the shoulder at about 524 nm were shown to be characteristic for salinixanthin and directly comparable with the peaks at 458, 486, and 521 nm previously reported by Lanyi and Balashov (2008) (Fig. 4, inset). Similar results are also observed for the samples isolated in presence of detergent (data not shown).

## Discussion

Extremophile bacteria are an emblematic example of the high level of biodiversity that permeates our planet. They succeeded in the challenge to evolve colonizing prohibitive environments. *S. ruber* is a magistral example of this adaptation, reaching such a level of specialization that high-salt environments, such as saltern crystallizer ponds, represent the only actual conditions where it optimally grows. This bacterium is even more peculiar if we consider that its habitat was assumed to be a large prerogative of Archaea species, typically specialized to these niches. In this sense, as also many other extremophile prokaryotes, such as, for example, *Thermus thermophilus* (Oshima and Imahori 1974; Farci et al. 2018a), *Deinococcus radiodurans* (Farci et al. 2016, 2018b) and *Haloferax volcanii* (Allers and Ngo 2003; Rodrigues-Oliveira et al. 2019), S. *ruber* represents an interesting model organism to study the evolution mechanisms in hypersaline habitats (Oren 2008, 2013; Antón et al. 2008; González-Torres and Gabaldón 2018), particularly with respect to their cell envelopes, the first forefront with the environment. Similarly to what is also observed in other extremophiles, such as *T. thermophilus* and *H. volcanii*, the levels of specialization reached by this organism are such that a high environmental specificity was reached (i.e., requiring at least 150 g salt/L for growing; (Antón et al. 2002)), making impossible for it to survive in more ordinary conditions.

Here, we have investigated composition and properties of the *S. ruber* cell envelope, finding it not only characterized by the presence of the XR system, as extensively reported in literature and also observed in our MS analysis (data not shown), but also by the presence of another highly represented protein characterized by the OMP_β-barrel domain (Table 1).

This protein was isolated by a mild approach that allowed a significant retention of native lipids. Together with a structural unbalance between large hydrophilic pores and much less representative hydrophobic surfaces, the retention of lipids might explain the unusual solubilization behaviour of these porin-like proteins (Welte et al. 1995). Moreover, the tendency to form transient and reversible aggregates, especially for short timeframes and before precipitation occurrence, might be an important strategy to warranty an efficient functionality in a crowd OM facing with a hypertonic environment (Saad et al. 2017). Considering the typical porin features suggested by the presence of the OMP_β-barrel domain, its localization in the outer membrane, and the co-presence of the Ton-B dependent receptor in the cell envelope samples, we called this protein OMPb-like protein (Table 1). Preliminary analysis were further supported by AI structural prediction using AlphaFold, identifying it as consisting of a 8 stranded β-barrel organization with dimensions comparable to those observed by an independent cryo-EM visualization (Fig. 3). Consequently, a question arises whether the main presence of this OM protein might have a generic functional mean limited to the difficult homeostatic maintenance under hypersaline conditions or might be also co-functional to the movement of positive charges associated with the XR proton pumping (Checover et al. 1997; Fu et al. 2013).

Several reports tried to elucidate the physiological implications related to both, the trafficking and the nutrients uptake under hypersaline conditions, as well as the proton gradient formation with respect to the charge balance during XR function (Oren et al. 2002; Balashov et al. 2005). In fact, in presence of light and hypersaline conditions, hence under operative conditions, a ∆pH and ∆[S] are maintained between the cytoplasm and the periplasm. This gradient is proportional to the light intensity and the salinity of the environment (Oren et al. 2002; Balashov et al. 2005). Under these conditions, an efficient porin system able to buffer these fluctuations would be pivotal in keeping charge neutrality. From the XR point of view, such a system would dump the effect of a transient unbalance of charges associated to an increased light-driven ∆pH with respect to the decrease in ∆pH associated to the ATP synthesis. In both cases, the presence of this protein would help in rationalizing the ion trafficking through a steady-state process that is directly related to the levels of external environmental salinity and light intensity, eventually allowing to maintain the cell homeostasis. The isolated porin might find its functional reason in this context, also supported by the presence of carotenoids. These cofactors could be crucial in contrasting the oxidative stress associated to an OM exposed to stressful conditions while sensing the light intensity and comparably with the XR activity acting proportionally. Accordingly, the findings presented in this report suggest a co-functionalization of the *S. ruber* cell envelope by the two systems, the OMPb-like protein and the XR, with the first having a dual function of stabilizing both, the fluctuation caused by the hypersaline conditions and the phototrophic activity. Further structural and functional studies are in progress to elucidate the basis of these mechanisms to finally test this complex hypothesis.

## Supplementary Information

Below is the link to the electronic supplementary material.

ESM 1 (PDF 336 KB)

## Data Availability

The original contributions presented in the study are included in the article/supplementary material, further inquiries can be directed to the corresponding author/s.
