# Supplementary material for: Isolation and characterization of a main porin from the outer membrane of Salinibacter ruber
Source: J Bioenerg Biomembr. 2022 Oct 13;54(5-6):273–81. doi: 10.1007/s10863-022-09950-7 (PMC9701654; doi:10.1007/s10863-022-09950-7)
Supplement: Supplementary file 1 — (PDF 336 KB) [file 10863_2022_9950_MOESM1_ESM.pdf]

Sup. Figure 1

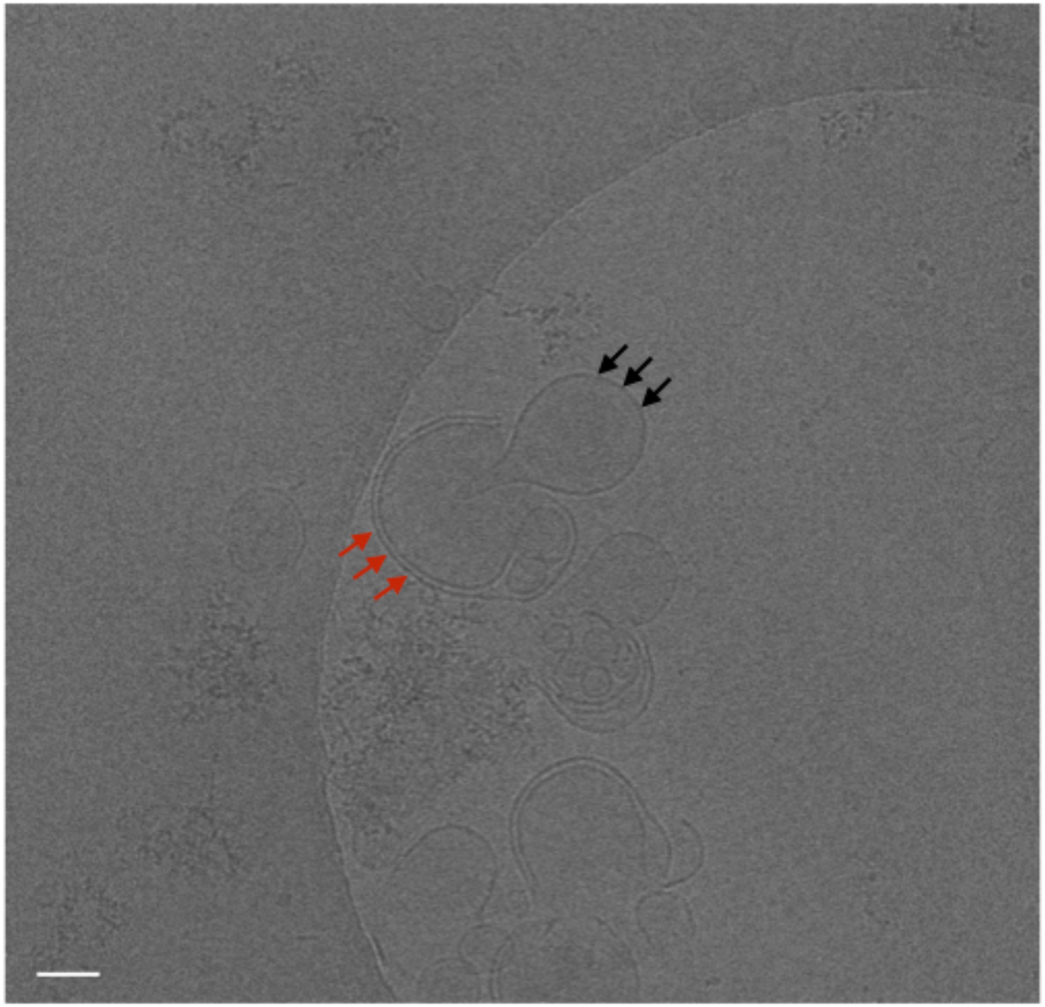

Supplementary Figure 1 - cryo-EM micrograph of the isolated cell envelopes. A typical grid field of the isolated cell envelopes after lysozyme digestion visualized by cryo-EM. The membranes appear as typical rounded fragments (red arrows) associated with vesicles-like structures (black arrows). A 100 nm scale bar is indicated.
